# Time Perspective Biases Are Associated With Poor Sleep Quality, Daytime Sleepiness, and Lower Levels of Subjective Well-Being Among Older Adults

**DOI:** 10.3389/fpsyg.2018.01356

**Published:** 2018-08-24

**Authors:** Michael Rönnlund, Maria G. Carelli

**Affiliations:** Department of Psychology, Umeå University, Umeå, Sweden

**Keywords:** time perspective, sleep quality, daytime sleepiness, deviations from a balanced time perspective, older adults

## Abstract

This study examined the extent to which individual differences in time perspective, i.e., habitual way of relating to the personal past, present, and future, are associated with sleep quality and daytime sleepiness in a sample of older adults. The participants (*N* = 437, 60–90 years) completed the Karolinska Sleep Questionnaire (KSQ), a the Swedish version of the Zimbardo Time Perspective Inventory (S-ZTPI), and two ratings of subjective well-being (SWB) (life satisfaction, happiness). Based on established relationships between dimension of time perspective and other variables (e.g., depression) and relations between negative retrospection (rumination) and negative prospection (worry) in prior studies, we expected higher scores on Past Negative and Future Negative to be linked to poor sleep quality and (indirectly) increased daytime sleepiness. Moreover, we examined the possibility that variations in perceived sleep and sleepiness during the day mediates the expected association between an aggregate measure of deviations from a so called balanced time perspective (DBTP) and SWB. In regression analyses controlling for demographic factors (age, sex, and work status), higher scores on Past Negative and Future Negative predicted poorer sleep quality and higher levels of daytime sleepiness. Additionally, most of the association between time perspective and daytime sleepiness was accounted for by individual differences in sleep quality. Finally, structural equation modeling yielded results consistent with the hypothesis that variations in sleep mediate part of the negative relationship between DBTP and SWB. Given that good sleep is essential to multiple aspects of health, future studies evaluating relationships between time perspective and adverse health outcomes should consider sleep quality as a potentially contributing factor.

## Introduction

Sleep serves many homeostatic processes and is essential for physical health, cognitive performance, and socio-emotional functioning (e.g., Beattie et al., 2015; Scullin and Bliwise, 2015; Patrick et al., 2017). One important aspect of sleep is the extent to which one perceives it to be free from disturbances, i.e., sleep quality. Interindividual differences in sleep quality may reflect a variety of situational or environmental factors, but, still, measurements of self-reported sleep tend to exhibit considerable stability over time (Knutson et al., 2006) and moderate heritability (e.g., Barclay et al., 2010). A potential association with dispositional factors, including dimensions of personality (e.g., Duggan et al., 2014; Gurtman et al., 2014) are therefore of interest. In the present study, we examined sleep quality and daytime sleepiness in relation to time perspective, a dispositional factor that attracted much recent research interest, but for which prior knowledge of relations to sleep is very limited.

### Time Perspective

Time perspective refers to the relative focus and valence, we assign to past, present, and future time frames (Zimbardo and Boyd, 1999). Temporal biases in form of an over-focus on some particular temporal frame or attitude are assumed to act as a disposition that exert an enduring influence on feelings, thoughts, and behaviors (Zimbardo and Boyd, 1999). The Zimbardo Time Perspective Inventory (ZTPI; Zimbardo and Boyd, 1999) is a widely used to capture individual differences in time perspective. This inventory encompasses five subscales: (1) Past Negative, which reflects a generally negative view of the past, (2) Past Positive, which captures a warm and nostalgic view of the past, (3) Present Hedonistic, which involves immediate pleasure seeking with little consideration of future consequences, (4) Present Fatalistic, which reflects a helpless attitude toward the present, where present behavior is considered as irrelevant to future costs or benefits, and, finally, (5) Future, which reflects a broad orientation toward the future that involves optimism and striving for future goals and rewards.

The unitary future dimension is of some concern as it focuses mainly of positive views and some people will focus on the future albeit bearing negative expectations of things to come. An extension of the original framework was therefore made in the development of the Swedish version of the ZTPI (S-ZTPI; Carelli et al., 2011). More specifically, the original Future dimension was replaced by two subscales: Future Positive, largely identical to the original Future and Future Negative scale, reflecting an aversive view of the future, mainly based on new items, much in line with the distinction between Past Positive and Past Negative. Empirical support of the differentiation between positive and negative aspects of a future time perspective includes a strong association of scores on Future Negative but not Future Positive (or Future) to use of maladaptive coping strategies (e.g., denial and substance use) in adolescents (Blomgren et al., 2016). Participants scoring above the cutoff for mild anxiety on Beck’s Anxiety Inventory moreover presented higher scores than non-anxious participants on Future Negative, while the groups did not differ on Future Positive (Åström et al., 2014). Moreover, level of perceived stress was strongly associated with Future Negative but unrelated to Future Positive and in common with Future Negative associated with *COMT* val158Met polymorphism (Rönnlund et al., 2018). Thus, differentiating positive and negative aspects of a future time perspective appears to be important to account for variance in behaviors and forms of mental ill-health.

A growing body of studies support the assumption that variations in time perspective (i.e., other than those pertaining to the future positive vs. negative distinction) are predictive of aspects of mental health (Stolarski et al., 2015). Different time perspective biases also appear to be characteristic of different disorders. For example, individuals who reported using drugs showed an overly focus on aspects of the present (Keough et al., 1999), whereas individuals with depressive disorder scored high on Past Negative in particular (Oyanadel and Buela-Casal, 2014).

Despite a need to consider specific dimensions of time perspective, a summary of biases (or deviations) from a proposed optimal ZTPI score profile has in addition proven to be useful. Researchers in the field devised a measure or index of such biases, referred to deviations from to a balanced time perspective (DBTP; Stolarski et al., 2011). DBTP is computed as an aggregate difference between an individual’s ZTPI score profile and an ideal (“balanced”) score profile, characterized by high scores on Past Positive, moderately high scores on Present Hedonistic and Future, low scores on Past Negative and Present Fatalistic (Stolarski et al., 2011). Importantly, DPTP showed a substantial (inverse) association with measures of subjective well-being (SWB), including ratings life satisfaction (e.g., Zhang et al., 2013), even when traditional personality factors were adjusted for (Stolarski and Matthews, 2016). More research seems to be needed to specify the mechanisms behind the well-established DBTP-SWB association, though. One factor that has been linked to life satisfaction is sleep quality (e.g., Zhi et al., 2016). In turn, sleep quality is, we argue, likely influenced by one’s temporal perspective, rendering sleep a plausible candidate as a mediator of the relationship between time perspective and SWB.

### Time Perspective and Sleep

There are several reasons to expect that time perspective is a factor behind between-person differences in sleep quality. First, two of the S-ZTPI dimensions, Past Negative and Future Negative, are conceptually linked to processes that were associated with sleeping difficulties in prior studies, namely rumination (e.g., Kirkegaard Thomsen et al., 2003; Yeh et al., 2015) and worry (e.g., Watts et al., 1994; Yeh et al., 2015). Defined broadly, rumination refers to repetitive thoughts arising with no direct cuing in response to an interruption of subjective goals (Martin and Tesser, 1996). Ruminative thinking typically concerns events that involved a perceived loss or failure, and, thus, often past-focused (cf. Past Negative; Carelli et al., 2015). By contrast, worry is conceptualized as repetitive thoughts primarily concerned with future events and with uncertainty (Segerstrom et al., 2000) a form of prospection involving negative expectations that should captured Future Negative (Carelli et al., 2015). Thus, even though rumination and worry have been regarded to share many features, a difference in regard to temporal content, i.e., concerned mainly with past vs. future events, is a distinguishing factor (Watkins et al., 2005) and both processes were linked to sleeping problems. Worry and rumination are furthermore regarded as core processes in development and maintenance of anxiety and depressive disorders (e.g., Evoy et al., 2013), in which sleeping problems are highly characteristic. Hence, a propensity to adversity toward the past and to host negative expectations of the future should be expected to be associated with sleeping problems, in turn increasing daytime sleepiness.

Despite the central role of sleep to maintain mental and physical health only one prior study that we know of, examined sleep quality in relation to time perspective, even though it should be acknowledged that a couple of studies considered relations to variations in diurnal preference or chronotype (e.g., Nowack and van der Meer, 2013; Stolarski et al., 2013; Milfont and Schwarzenthal, 2014). The only extant study of sleep quality (Vranesh et al., 1999) was a short report based on student sample (*n* = 135) with limited discussion of the results. In line with the predictions above, higher scores on Past Negative were significantly associated with sleeping problems. So were also scores on Present Fatalistic and Present Hedonistic. As noted by the authors high scores on the present-scales have been linked to less healthy life style choices, e.g., smoking and use of alcohol (Keough et al., 1999) known to have a detrimental effect on sleep (e.g., Ebrahim et al., 2013), which could possibly account for the latter associations, even though no data on smoking and alcohol use were reported to support these assumptions. Surprisingly, a higher score on the two remaining subscales, i.e., Past Positive and Future, were associated with sleeping problems. These patterns served as a basis for the authors to conclude that *any* type of time related focus may cause sleep-related problems. The analyses were restricted to bivariate associations of ZTPI dimensions and sleep quality scores, which precludes conclusions regarding what dimensions were the most prominent predictors of sleep. A multivariate analyses is motivated by the small to moderate inter-correlations of the ZTPI dimensions (e.g., Zimbardo and Boyd, 1999; Carelli et al., 2011). Of final concern, the version of the ZTPI used in the former study lacked the future negative scale, which we, as noted, expect to be a correlate of sleeping problems.

### The Present Study

Given the scant evidence regarding the association between time perspective and sleep the major of aim of the present study was to examine associations between time perspective sleep quality/daytime sleepiness. The present study involved an older (≥60 years) population. Whereas, we see no obvious reason to expect the association between time perspective dimensions, sleep-related factors, and life satisfaction to be substantially altered by adult age, sleep-related problems may be regarded to be of particular concern in groups of more fragile individuals, including elderly. Indeed, research suggests that outcomes of special relevance to old age may be moderated or even caused by sleep-related problems, including falls (Hayley et al., 2015) and cardio-vascular disease (Newman et al., 2000). Some studies also indicate that sleep complaints are associated with unfavorable cognitive outcomes (e.g., deficits in working memory, attentional set shifting, abstract problem solving; for a review, see Lo et al., 2016 for a review) and adults reporting excessive daytime sleepiness (and fatigue) may be at elevated risk of cognitive decline (Keage et al., 2012; Yaffe et al., 2014).

Motivated by links to negative thought processes and affective states/disorders linked to sleeping problems, higher scores on Past Negative and Future Negative were hypothesized to be the major predictors of poorer sleep quality. The aforementioned S-ZTPI dimensions were moreover expected to predict daytime sleepiness via poorer sleep quality. Motivated by links to less health behaviors and depressive symptoms (Desmyter and De Raedt, 2012) higher Present Fatalistic was also expected to be associated with sleep-related problems. A secondary aim was to test the viability of a hypothetical model according to, which the expected link between DBTP and SWB (life satisfaction, happiness) is (at least in part) mediated by variations in sleep quality and daytime sleepiness.

## Materials and Methods

### Participants

The data were collected as part of the Betula prospective cohort study (Nilsson et al., 1997, 2004) that was based on random sampling of participants from the population registry in Umeå municipality, Sweden. The present study involved data for participants in two subsamples (Samples 1 and 3) that were collected at the sixth measurement occasion (2013–2014), when the questionnaire concerning time perspective (S-ZTPI) was added to the battery.

A total of 437 participants, 204 men and 233 women, aged 60, 65, 70, 75, 80, 85, or 90 years at date of the assessment, met the inclusion criteria. These were: (1) having completed at least 80% of S-ZTPI item ratings (explained in more detail below), (2) Mini-Mental State Examination (Folstein et al., 1975) score ≥24, and (3) having responded to ≥80% of relevant ratings in the Karolinska Sleep Questionnaire (KSQ). For participants with a minor number of partially missing values (<20% on the S-ZTPI and KSQ) values were imputed, using the Expectation Maximum Method (Dempster et al., 1977). To provide an idea of the extent of missingness, 89.5% of the participants had responded to all 64 S-ZTPI items, 8% had missed one item and 2.5% missed between 2 and 9 items. KSQ ratings showed a similar pattern in regard to missingness with a majority of those with missing values missing only one item. A comparison of analyses with or without (i.e., list-wise deletion of missing data) imputation shown negligible differences.

The mean age of the sample was 70.2 (*SD* = 7.7). Self-reported work status (being employed, running own farm, or own company considered as working) indicated that a majority of the participants (*n* = 300 or 68.6%) were retired, while 137 individuals (31.4%), mainly in the 60 to 65-year groups, were still working. Based on the assumption that work status may affect sleeping patterns and sleep quality/daytime sleepiness, this factor was considered as a covariate in the analyses.

### Instruments

#### Karolinska Sleep Questionnaire

The version of the KSQ (Åkerstedt et al., 2008) used involves global ratings of sleep and questions regarding the duration and timing of nocturnal sleep and ratings of more specific problems with sleep onset, nocturnal sleep, awakening, and sleepiness during the day (e.g., “*difficulties falling asleep*,” “*not feeling restored at time of awakening*,” “*sleepy during leisure time*”). The latter were in focus in the present study. For each of items, the participant is requested to rate the frequency of occurrence of the described problem during the last 3 months, on a six-point scale; “Never” (coded as 0), “Rarely” (coded as 1), “Sometimes (several times per month)” coded as 2, “Often (one to two time per week)” coded as 3, “Most of the time (three to four times/week)” coded as 4, and “Always (five times per week or more)” coded as 5. The ratings have been summarized into several indexes: a sleep quality/insomnia index, a non-restorative sleep/awakening index, a sleep apnea index, and a daytime sleepiness index. In the present study, questions pertaining to sleep quality (four items), non-restorative sleep (three items), and daytime sleepiness (five items) were considered. In regard to internal consistency (Cronbach’s α), values above the cutoff for acceptable consistency (>0.70; Nunnally, 1978) were reported for the sleep quality index, the non-restorative index (Nordin et al., 2013) as well as the daytime sleepiness index (Eriksen and Kecklund, 2007). Validity evidence for the sleep quality index and the non-restorative sleep/awakening index included associations with physiological sleep measures (Westerlund et al., 2016); stage 2 and slow-wave sleep predicted worse and better sleep quality and slow-wave sleep predicted less perceived restoration.

#### Swedish Zimbardo Time Perspective Inventory

Swedish Zimbardo Time Perspective Inventory (Carelli et al., 2011) consists of 64 items, the 56 items in the ZTPI (Zimbardo and Boyd, 1999) and eight extra items for the Future Negative scale. Each item is a statement reflecting one of six temporal dimensions: Past Positive (e.g., “Familiar childhood sights, sounds, smells often bring back a flood of wonderful memories”), Past Negative (e.g., “Painful past experiences keep being replayed in my mind”), Hedonistic Present (e.g., “I believe that getting together with one’s friends to party is one of life’s important pleasure”); Present Fatalistic (e.g., “Fate determines much in my life”); Future Positive (e.g., “When I want to achieve something, I set goals and consider specific means for reaching those goals”), Future Negative (e.g., “The future contains too many boring decisions that I do not want to think about”). The participant is requested to rate each statement in regard to how characteristic it is of his/her own view on five-point Likert scale. This ranges from “very uncharacteristic” (coded as 1) to “very characteristic” (coded as 5). S-ZTPI has demonstrated adequate reliability with internal consistency ranging from 0.70 for the Future Positive scale to 0.84 for the Past Negative scale and test-retest reliability at 0.60 to 0.85 (Carelli et al., 2011). Evidence in regard to convergent validity included associations with the General Decision Making Styles scale (Scott and Bruce, 1995) and the Barratt Impulsiveness Scale (Patton et al., 1995) with correlations of the S-ZTPI dimensions in expected directions (Carelli et al., 2011).

Apart from score on the separate subscales, we computed Deviations from a balanced time perspective based on the six S-ZTPI subscales (referred to as DBTP-E to denote a difference from the formula for DBTP based on the original ZTPI) in accord with prior studies (Stolarski et al., 2011; Rönnlund et al., 2017):

$$\sqrt{\begin{array}{l} {\left(\text{oPN-ePN}\right)}^{2}+{\left(\text{oPP-ePP}\right)}^{2}+{\left(\text{oPF-ePF}\right)}^{2}+\\ {\left(\text{oPH-ePH}\right)}^{2}+{\left(\text{oFP-eFP}\right)}^{2}+{\left(\text{oFN-eFN}\right)}^{2} \end{array}},$$

where o = optimal score and e = empirical (i.e., observed) score. In line with previous studies ideal values for the S-ZTPI subscales were set to: oPN = 1.95, oPP = 4.6, oPF = 1.5, oPH = 3.9, oF/oFP = 4.0, and oFN = 1.8. The specific values were based on percentile ranks in a large cross-cultural database of data on the original version of ZTPI (Stolarski et al., 2011); the value for FN was set at 10th percentile, in analogy with the proposed optimal scores for PN and PF (Rönnlund et al., 2017).

### Subjective Well-Being

Two ratings were included as indicators of SWB. The first was a rating of global life satisfaction; “Taking everything in life into account, how satisfied with your life are you?” made on a scale from 0 (extremely unsatisfied) to 10 (extremely satisfied). The second was a rating of happiness (“Taking everything into account, how happy would you say you are?”) on a scale from 0 (extremely unhappy) to 10 (extremely happy). Similar single 11-point ratings of happiness (Abdel-Khalek, 2006) and life satisfaction (Cheung and Lucas, 2014) showed good convergent validity with more comprehensive measures such as the Oxford Happiness Inventory (Argyle et al., 1995) and the Satisfaction with Life Scale (Diener et al., 1985).

### Statistical Methods

We used exploratory factor analysis (Principal Axis Factoring with Promax Rotation) to examine the dimensionality of the included KSQ items. This was motivated by the fact that two of items concerned sleepiness during work were omitted from the analyses (i.e., as a majority of participants were retired). A minor number of missing values (<3% for each item) that remained for the other 10 items, apparently missing at random, were first imputed, using the Expectation-Maximization algorithm (Dempster et al., 1977). The resulting factor scores were used in subsequent regression analyses (i.e., as the dependent variables), in which the predictors were entered in a hierarchical fashion with forced entry of the variables (e.g., demographic variables in step one, time perspective dimensions in step two). In order to provide a check of potential concerns in regard to multicollinearity, variance inflation factors, and values for tolerance were considered in relation to common threshold values (Menard, 1995). In order to examine the hypothesis that the sleep factors mediate the relationship between the summary measure of time perspective biases (DBTP) and a SWB factor, we set up a mediational model and examined the direct and indirect effects. The three items ratings with highest loading on factor 1 (poor sleep quality) in previous analyses (i.e., “difficulties falling asleep,” “repeated awakenings with difficulties falling asleep again,” and “not feeling restored upon awakening”) were considered as indicators of a factor reflecting poor sleep quality. The two ratings with high and unique loadings on the second factor (“involuntary periods of sleep during leisure time” and “need to fight against sleep”) served as indicators of a latent daytime sleepiness factor (see section “Results”). Finally, the two SWB ratings (Life satisfaction; LiSAT and Happiness; Happy) were used as indicators of a latent SBW factor. The model further included a path from insomnia to daytime sleepiness. To evaluate model fit, two indexes were considered: the Comparative Fit index, CFI (values >0.95 taken to indicate good model fit (Hu and Bentler, 1999)) and Root Mean Squared Error of Approximation, RMSEA (with a value of <0.06 indicative of good model fit and <0.08 indicative of reasonable fit (Hu and Bentler, 1999). Bootstrap analyses, involving 200 bootstrap samples were performed to test the significance of the direct and indirect effects/generate bias-corrected confidence intervals (95%). The analyses were conducted using IBM SPSS 23.0 and AMOS.

## Results

### Factor Analysis of KSQ Items and Data Reduction

**FIGURE 1:**
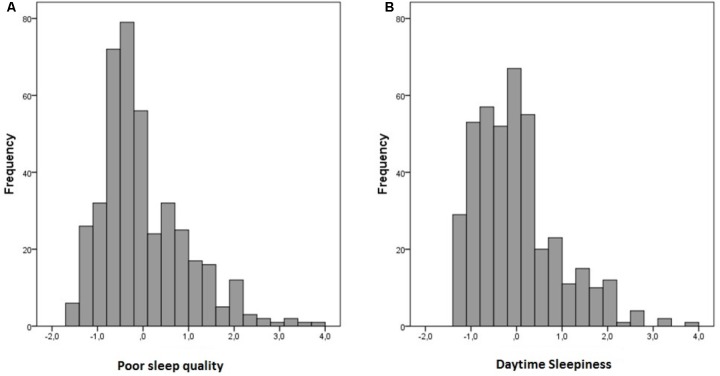
Frequency distribution of Poor Sleep Quality **(A)** and Daytime Sleepiness factor scores **(B)**.

An exploratory factor analysis, using Principal Axis Factoring with Promax rotation, was performed on the 10 KSQ ratings. Kaiser-Meyer-Olkin measure of sampling adequacy (0.85) was sufficient (>0.70) and Bartlett’s test of sphericity was significant (*p* < 0.001). Based on the Kaiser rule (eigenvalue > 1) and inspection of the scree plot (elbow rule), two factors, accounting for 41.0 and 11.3% of the variance, respectively, were extracted. The pattern matrix loadings for the first factor were high (>0.60) for six of the items. These items were all from the sleep quality index or the non-restorative sleep index, with highest loadings for “difficulties falling asleep,” “repeated awakenings,” and “not refreshed upon awakening.” None of the six items exhibited a substantial loading (≥0.30) on factor 2. By contrast, high loadings (>0.70) on the second factor were observed for two items; a third rating showed a substantial loading 0.48 on factor 2, but also a cross-loading above 0.30 on factor 1; all other item loadings were below 0.30. These two ratings were from the daytime sleepiness index: “involuntary periods of sleep during leisure time” and “need to fight against sleep.” Thus, we interpreted the first factor to reflect poor sleep quality, including difficulties with sleep onset and sleep maintenance, and the second factor to reflect daytime sleepiness. We used a regression option to generate factor scores to be used in subsequent analyses. The frequency distribution of the two resulting scores are shown in **Figure 1A** (Poor Sleep Quality) and **Figure 1B** (Daytime Sleepiness).

As should be expected in a non-clinical sample, distributions of the factor scores exhibited a slight positive skew (skewness = 1.09 for Poor Sleep Quality and 1.07 for Daytime Sleepiness), but was not deemed to warrant transformation (transformation and/or application of non-parametric statistics yielded highly similar results as those reported here). As expected, the two sleep factor scores were positively correlated (*r* = 0.55, *p* < 0.001).

### Zero-Order Correlations and Descriptive Statistics

The Sleep Quality and Daytime Sleepiness factor scores and the variables considered as predictors were next submitted to a correlational analysis. The resulting values and descriptive statistics (M, SD) for the set of study variables are presented in **Table 1**.

**Table 1:** Bivariate associations of variables and descriptive statistics.

Variable	*M*	*SD*	1	2	3	4	5	6	7	8	9	10	11	12
(1) Age	70.3	7.6	1											
(2) Sex (*m* = 0, *f* = 1)	–	–	0.02	1										
(3) Work status (*w* = 1, not *w* = 0)	–	–	−0.59*	−0.07	1									
(4) Past Positive	3.58	0.51	0.09	0.02	−0.04	1								
(5) Past Negative	2.25	0.59	0.18*	−0.02	−0.14*	−0.23*	1							
(6) Present Hedonistic	2.90	0.44	0.05	0.02	0.03	0.26*	0.16*	1						
(7) Present Fatalistic	2.49	0.53	0.30*	0.10*	−0.16*	0.07	0.40*	0.37*	1					
(8) Future Positive	3.26	0.42	−0.01	−0.07	−0.06	0.14*	0.17*	−0.03	−0.11*	1				
(9) Future Negative	2.51	0.55	−0.11*	0.08	−0.03	−0.05	0.63*	0.17*	0.42*	0.31*	1			
(10) PSQ score	0.00	1.00	0.01	0.12*	0.08	−0.01	0.27*	−0.02	0.12*	0.06	0.34*	1		
(11) DTS score	0.00	1.00	−0.07	0.00	−0.19*	−0.02	0.29*	0.08	0.10*	0.06	0.32*	0.55*	1	

*SQ, Sleep Quality; DTS, Daytime Sleepiness, PSQ, Poor Sleep Quality, ^∗^p < 0.05.*

**Table 2:** Summary of regression analyses of Poor Sleep Quality and Daytime sleepiness factor scores.

		Poor sleep quality				Daytime sleepiness–analysis 1				Daytime sleepiness–analysis 2			
				
	Variable	Δ*R*^2^	Tot. *R*^2^	β^a^	*p*	Δ*R*^2^	Tot. *R*^2^	β	*p*	Δ*R*^2^	Tot. *R*^2^	β	*p*
Step 0/1	PSQ	–	–	–	–	–	–	–	–	0.304^∗∗^	0.304	0.551	0.000
Step 1/2	Age	0.027^∗^	0.027	0.088	0.135	0.038^∗∗^	0.038	0.067	0.50	0.024^∗∗^	0.327	0.019	0.695
	Sex (female = 1)			0.126^∗∗^	0.009			0.011	0.820			−0.058	0.149
	Work status			0.142^∗∗^	0.046			0.226^∗∗^	0.000			0.149^∗∗^	0.003
Step 2/3	Past Positive	0.129^∗∗^	0.156	0.078	0.121	0.124^∗∗^	0.166	0.058	0.247	0.039^∗∗^	0.367	0.020	0.646
	Past Negative			0.163^∗∗^	0.010			0.214^∗∗^	0.001			0.135^∗^	0.014
	Present Hedonistic			−0.097	0.055			0.028	0.567			0.075	0.086
	Present Fatalistic			−0.044	0.442			−0.085	0.134			−0.064	0.199
	Future Positive			−0.077	0.126			−0.085	0.090			−0.048	0.277
	Future Negative			0.289^∗∗^	0.000			0.248^∗∗^	0.000			0.109	0.055

*^a^Values are for first entry, ^∗^p < 0.05, ^∗∗^p < 0.01. PSQ, Poor Sleep Quality.*

Of the S-ZTPI dimensions, Past Negative and Future Negative were positively correlated with poorer sleep quality (*r* = 0.27 and *r* = 0.34, respectively, *p* < 0.001) and with scores on the daytime sleepiness factor (*r* = 0.29 and *r* = 0.32, *p* < 0.001). Present Fatalistic score was additionally exhibited a positive association with poorer sleep quality and daytime sleepiness (*r* = 10–0.12, *p* < 0.05). In order to control for the possibility that the patterns of associations differed across age split samples [young-old, 60–70 (*n* = 267) vs. old-old; 75–90 years (*n* = 170)] were inspected. Highly similar patterns to those observed for the entire sample applied to the separate age groups were obtained, with significant correlations between Past Negative, Future Negative and scores on the sleep-related factors.

### Regression Analyses of Sleep Quality and Daytime Sleepiness

To estimate the relative and total contribution of the entire set of predictors (**Table 1**), hierarchical regression analyses were conducted. In the analysis of Sleep quality, and in the first analysis (Analysis 1) of Daytime sleepiness scores, the demographic variables (age, sex, and work status) were entered in a first step (i.e., as a block) with forced entry of the predictors. In the second and final step, scores on the six S-ZTPI dimensions were entered (Analysis 1). To examine the extent to which the predictors were related to Daytime Sleepiness over and beyond Sleep Quality, a second analysis (Analysis 2) of the Daytime sleepiness included scores on the Poor Sleep Quality factor as a predictor in the first step. Apart from this, order of entry of variables was the same as in prior analyses, i.e., demographic factors (step 2) followed by the S-ZTPI dimensions (step 3). Variance Inflation factors were no larger than 2.1 and tolerance values were all >0.49 indicating no multicollinearity problems across the models. The results of the three regression analyses are summarized in **Table 2**.

Beginning with Poor Sleep Quality, the demographic variables accounted significant variance (*R*^2^ = 0.027). Female sex (β = 0.126, *p* = 0.02) and working (β = 0.142, *p* < 0.01) were significant predictors in step 1. In step 2, the time perspective dimensions added significant variance (Δ*R*^2^ = 0.129, *p* < 0.001). Past Negative (β = 0.163, *p* = 0.01) and Future Negative (β = 0.289, *p* < 0.001) were the unique predictors.

The corresponding analysis of the Daytime Sleepiness scores (Analyses 1) revealed a similar pattern of results, with each block contributing with significant variance at steps 1–3. Within the demographic block, working was associated with higher daytime sleepiness scores (β = 0.226, *p* < 0.001). The S-ZTPI dimensions accounted for a significant amount of variance in step 2, with Past Negative (β = 0.214, *p* = 0.001 and Future Negative (β = 0.248, *p* < 0.001) as the significant predictors.

In the second analyses of Daytime Sleepiness scores (Analyses 2), Sleep Quality entered in the first step was a strong predictor (β = 0.552, *p* < 0.001) in step 1. In step 2, work status was a significant predictor (β = 0.149, *p* < 0.001, as in the first analyses. Finally, in step 3, the S-ZTPI dimensions were significant as a block (Δ*R*^2^ = 0.039, *p* < 0.01). As in the first analyses (Analysis 1), Past Negative was a significant predictor of Daytime Sleepiness (β = 0.135, *p* < 0.014), with a tendency (*p* = 0.055) in the same direction for Future Negative.

### Deviations From a Balanced Time Perspective and Sleep Factors in Relation to SWB

**FIGURE 2:**
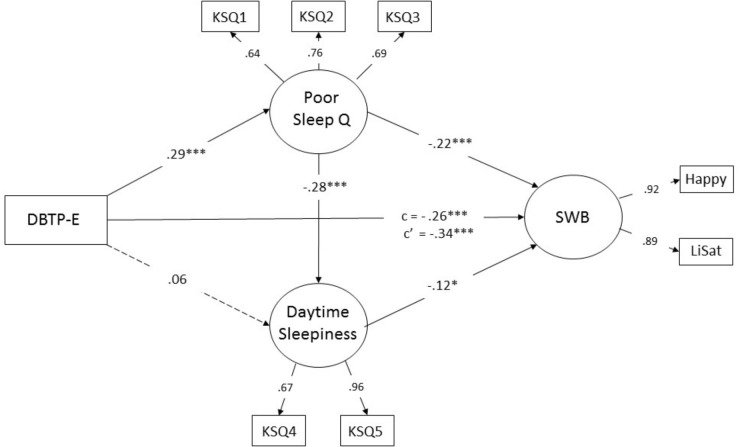
Structural model of hypothetical relationships between of deviations from a balanced time perspective (DBTP-E), sleep factors (Poor Sleep Quality and Daytime Sleepiness), and subjective well-being (SWB). Values are standardized coefficients (β-values). *c* is the direct (unmediated) standardized effect of DBTP-E on SWB. *c*’ represents the total standardized effect ^∗^*p* < 0.05, ^∗∗∗^*p* < 0.001.

As outlined previously, we aimed to test a model involving a measure of deviations from a balanced time perspective (DBTP-E; see section “Materials and Methods”) as a predictor of SWB with potential direct and hypothesized indirect effects on SWB via poor sleep quality with possible influences through higher daytime sleepiness as well. A simplified version of the model, including standardized item-loadings and standardized regression coefficient for the hypothesized paths is depicted in **Figure 2**.

The model showed good/reasonable fit as judged by values for CFI (0.97) and RMSEA (0.072, 90% CI 0.05–0.09) respectively, χ^2^ (*df* = 15) = 49.2, *p* < 0.001. In line with the hypotheses, significant paths from DBTP-E to poor sleep quality (β = 0.29, *p* < 0.001) and from DBTP-E to SWB (β = -0.26, *p* < 0.001) are observed, with a non-significant positive value for the path from DBTP-E to daytime sleepiness (β = 0.06, *p* > 0.10), but as can be seen the presence of significant paths from DBTP-E to Sleep Quality and from the latter factor to Daytime Sleepiness (β = 0.28, *p* > 0.001) suggest an indirect effect of temporal biases on Daytime Sleepiness. Significant paths from poor sleep quality to SWB (β = -0.22, *p* < 0.001) as well as from Daytime Sleepiness to the SWB factor (β = -0.12, *p* = 0.04) were in addition observed.

The results of bootstrap analyses confirmed that the direct effects (i.e., except that that from DBTP-E to daytime sleepiness), were significant (*p* < 0.02). Importantly, the results indicated significant indirect effects of DBTP-E on Daytime Sleepiness (standardized effect 0.082, *p* = 0.004, tow-tailed) and of DBTP-E on SWB (-0.080, *p* = 0.011, two-tailed), as well as a significant indirect effect of poor sleep quality on SWB via increased daytime sleepiness (-0.033, *p* = 0.012). Whereas the foregoing results were consistent with the hypothesis that the sleep-related constructs mediated a significant part of the relationship between deviations from a balanced time perspective and SWB, it is warranted to point out that it was rather modest as judged from a comparison of the direct effect observed in this model (*c* = -0.26) with the total (unmediated) effect of DBTP-E on SWB (*c*’ = -0.34; yielding a ratio of direct-to-total effect of 0.76).

## Discussion

This study examined sleep quality and daytime sleepiness in relation to time perspective in a sample of older adults. A hypothetical model by which deviations from a balanced time perspective influences SWB via poor sleep was furthermore tested. Consistent with our primary hypotheses, the time perspective dimensions capturing an aversive orientations toward the past and the future, i.e., Past Negative (see also Vranesh et al., 1999) and Future Negative were related to poorer sleep quality. Of the two dimensions, Future Negative appeared to be the more prominent predictor of sleep quality as well as daytime sleepiness, providing another example of the important role of Future Negative to account for health-related factors and behaviors (Carelli et al., 2011; Åström et al., 2014; Blomgren et al., 2016; Rönnlund et al., 2017, 2018). As concerns daytime sleepiness, most of the variance in scores accounted for by the S-ZTPI dimensions appeared to be indirect, i.e., to reflect that the relationship was largely attributable to an influence on sleep quality. More specifically, entry of Sleep Quality in *a prior* step reduced the variance in Daytime Sleepiness accounted for by the S-ZTPI dimensions considerably. A small but significant portion of the variance remained, though, and Past Negative persisted as a significant unique predictor of daytime sleepiness even when sleep quality was adjusted for. A relation of Past Negative to factors that influence sleepiness or fatigue over and beyond nocturnal sleep (e.g., Desmyter and De Raedt, 2012; Oyanadel and Buela-Casal, 2014) may account for the significant prediction of the residual variance in daytime sleepiness.

In line with the prediction based on Vranesh et al. (1999), scores on Present Fatalistic moreover exhibited a significant positive association with sleeping problems and was associated with daytime sleepiness as well. However, the associations were small and not observed in the analyses including the other S-ZTPI dimensions as the predictors. In contrast to the results in Vranesh et al. (1999) higher scores on Past Positive and Future Positive were *not* associated with increased sleeping difficulties or daytime sleepiness, neither as judged from the bivariate association nor results from multivariate analyses, though. The present results appear reasonable given relations of Past Positive or Future to health-promoting behaviors, for example medication adherence (Sansbury et al., 2014) and healthy life style habits, including less frequent use of alcohol and smoking (Keough et al., 1999). If anything, such findings have been served as a basis for expecting a negative association with sleep-related problems. Differences in age composition in the present sample and that in Vranesh et al. (1999) could of course be a factor and future studies need to examine the extent to which the present patterns, and those in Vranesh et al. (1999), generalize to younger adults.

A secondary aim was to examine the possibility that variations in sleep-related problems are a mediator of the expected association between deviations from an optimal S-ZTPI profile (DBTP or DBTP-E). A structural model with hypothetical direct and indirect links between time perspective the measure of DBTP, the sleep-related constructs and SWB yielded results that were largely consistent with the hypothesized model. As predicted, DBTP was positively related to the insomnia factor, indicating that the more an individual’s time perspective depart from the constellation of optimal S-ZTPI scores, the more likely one is to experience sleeping difficulties. The results were furthermore, consistent with an indirect effect of DBTP on daytime sleepiness, much in line with results from the regression analyses. Importantly, the results were also consistent with a hypothesis that part of the relationship between DBTP and SWB is mediated by sleep. Even though the indirect effect was rather small, and each of the three factors (i.e., DBTP, sleep quality, and daytime sleepiness) appeared to have a unique influence on SWB, it suggests at least, that poor sleep might be one of several factors contributing to the well-established relationship between and SWB (Zhang et al., 2013; Stolarski and Matthews, 2016). Hence, future studies aimed to study relationships between deviations from BTP and adverse health outcomes (e.g., depression; Oyanadel and Buela-Casal, 2014 and anxiety; Åström et al., 2014) should consider a potential role of sleeping problems to the associations.

As concern results pertaining to the demographic variables, the result of poorer sleep quality in women is consistent with prior studies (e.g., Mallon et al., 2002; Vitello et al., 2004). In the present study, age was not significantly related to sleep quality nor daytime sleepiness in line with some prior studies (e.g., Holfeld and Ruthig, 2014). The observation of a positive association between work status and insomnia and daytime sleepiness is consistent with findings of lowered odds of sleep disturbance in the post- compared with in the pre-retirement period, except when retirement was based on health-related problems (Vahtera et al., 2016), though. Taken together, the results in regard to associations of the demographic factors and aspects of sleep were largely consistent with those in prior studies.

### Limitations

Despite strengths including a comprehensive measurement of the variables and a relatively large and population-based sample, limitations of this study should be acknowledged. Importantly, the data were cross sectional. Based on the theoretical framework by Zimbardo and Boyd (1999), time perspective biases were hypothesized to influence sleep, but poor sleep could, in the long run (at least), certainly influence one’s time perspective. To provide a good test of the hypothesized causal links between time perspective biases, aspects of sleep, and SWB, longitudinal data are required. Second, self-report data on sleep are informative, but should ideally be accompanied with objective recordings of sleep and sleep behavior (i.e., actigraphy or polysomnography). Third, the total amount of variance in the sleep measures accounted for by the set of variables was rather modest. This could partly reflect the nature of the data(self-reports) but certainly implies that influence of a variety of additional variables needs to be considered to provide a full account of individual differences in sleep quality and daytime sleepiness. Finally, we failed to take another interesting aspect of sleep, namely chronotype (variations along a morningness to eveningness continuum) into account. Indeed, a solid body of research demonstrated a significant time perspective—chronotype association (Nowack and van der Meer, 2013; Stolarski et al., 2013; Milfont and Schwarzenthal, 2014). Whereas the main message of these studies are that present vs. future (positive) orientation is related to chronotype, Stolarski et al. (2013) found that DBTP was associated with morningness, in a young adult sample. Given evidence that chronotype is robustly associated with sleep quality (Juda et al., 2013; Vitale et al., 2015), it hence appears reasonable to take this factor into account to understand the link between time perspective, sleep-related outcomes, and SWB.

## Conclusion

This study demonstrated that time perspective biases, in particular a negative orientations toward the past and the future are related to poor sleep quality and daytime sleepiness in older adults. The analyses suggested that that DBTP, sleep quality and daytime sleepiness each contribute to lower SWB among older adults, but were also consistent with the idea that part of the negative relationship between DBTP and SWB is mediated by sleeping problems. Based on these observations, poor sleep quality is a factor to consider also when relationships between time perspective and adverse health outcomes are evaluated. Finally, in regard to practical implications, the link of sleep and time perspective biases may suggest that interventions with a potential to foster a more balanced time perspective, such as mindfulness-based interventions (Stolarski et al., 2016; Droit-Volet and Heros, 2017) might prove effective to reduce sleeping problems in older adults (see Black et al., 2015). Clearly, future work, ideally longitudinal studies, are required to disentangle the proposed relationships among time perspective, sleep-related variables, and well-being. Finally, future studies should consider additional sleep factors, such as in chronotype in this context, and consider the inclusion of objective sleep measures.

## Ethics Statement

This study was carried out in accordance with the recommendations of the regional ethic board in Umeå, Sweden that approved the study protocols. All subjects gave written informed consent in accordance with the Declaration of Helsinki.

## Author Contributions

MR performed the analyses and wrote the first draft of the paper. Both authors conceptualized the study and made critical revisions of the draft.

## Conflict of Interest Statement

The authors declare that the research was conducted in the absence of any commercial or financial relationships that could be construed as a potential conflict of interest.
